# *GmLecRlk*, a Lectin Receptor-like Protein Kinase, Contributes to Salt Stress Tolerance by Regulating Salt-Responsive Genes in Soybean

**DOI:** 10.3390/ijms23031030

**Published:** 2022-01-18

**Authors:** Yanzheng Zhang, Qingwei Fang, Jiqiang Zheng, Zeyang Li, Yue Li, Yuan Feng, Yingpeng Han, Yongguang Li

**Affiliations:** Key Laboratory of Soybean Biology of Ministry of Education China, Key Laboratory of Soybean Biology and Breeding (Genetics) of Ministry of Agriculture and Rural Affairs, Northeast Agricultural University, Harbin 150030, China; yanzhengzhang1992@163.com (Y.Z.); fqw2536901069@163.com (Q.F.); zhengsichen1226@163.com (J.Z.); lzy780058207@163.com (Z.L.); lywcc19990804@163.com (Y.L.); z1072338704@163.com (Y.F.)

**Keywords:** soybean, *GmLecRlk*, lectin receptor protein kinase, salt tolerance, RNA-seq

## Abstract

Soybean [*Glycine max* (L.) Merr.] is an important oil crop that provides valuable resources for human consumption, animal feed, and biofuel. Through the transcriptome analysis in our previous study, *GmLecRlk* (*Glyma*.*07G005700*) was identified as a salt-responsive candidate gene in soybean. In this study, qRT-PCR analysis showed that the *GmLecRlk* gene expression level was significantly induced by salt stress and highly expressed in soybean roots. The *pCAMBIA3300*-*GmLecRlk* construct was generated and introduced into the soybean genome by *Agrobacterium rhizogenes*. Compared with the wild type (WT), *GmLecRlk* overexpressing (*GmLecRlk*-ox) soybean lines had significantly enhanced fresh weight, proline (Pro) content, and catalase (CAT) activity, and reduced malondialdehyde (MDA) and H_2_O_2_ content under salt stress. These results show that *GmLecRlk* gene enhanced ROS scavenging ability in response to salt stress in soybean. Meanwhile, we demonstrated that *GmLecRlk* gene also conferred soybean salt tolerance when it was overexpressed alone in soybean hairy root. Furthermore, the combination of RNA-seq and qRT-PCR analysis was used to determine that *GmLecRlk* improves the salt tolerance of soybean by upregulating *GmERF3*, *GmbHLH30*, and *GmDREB2* and downregulating *GmGH3*.*6*, *GmPUB8*, and *GmLAMP1*. Our research reveals a new mechanism of salt resistance in soybean, which exposes a novel avenue for the cultivation of salt-resistant varieties.

## 1. Introduction

Soybean is an important economic and oil crop, but abiotic stress, such as saline–alkali stress, has a great impact on soybean yield. Although soybean is a moderately salt-tolerant crop, soybean yield can still be reduced by more than 50% in salinized land [1]. Therefore, mining salt-tolerant genes is important for improving soybean salt tolerance and cultivating new soybean varieties. All kinds of receptors located on the cell membrane or in plant cells can specifically recognize bioactive molecules and receive and process external information. The regulation of receptors is complex, including transcriptional, translational, and metabolic levels, and finally coordinates and controls plant growth and development. The translated protein in plant cells need a series of post-translational modifications, in which phosphorylation plays a key role in the process of cell life [2]. Protein phosphorylation is catalyzed by protein kinases, and a class of proteins similar to protein kinases, named receptor-like protein kinases (RLKs), are found in plants. According to the difference in the amino acid sequence of the extracellular domain of plant RLKs, they can be divided into six types: S-domain type [3], leucine-rich multiple sequence type (LRR) [4], epidermal growth factor-like type (EGF) [5], lectin-like type [6], tumor necrosis factor-like receptor type (TNFR) [7], and PR5-like type (PR5K) [8].

Lectin receptor protein kinases (LecRLKs) comprise a subfamily of the RLK family that consists of four domains: amino terminal signal peptide (N-terminal signal peptide), ligand binding domain (ligand-binding domain), single-channel transmembrane domain (single-pass transmembrane domain), and intracellular serine/threonine kinase domain (cytoplas-mic Ser/Thr kinase domain). LecRLKs contain a hydrophobic concave ligand binding domain that is mainly used to bind hydrophobic molecules, such as sugars, plant hormones, and cytokinins, and are important RLKs [9]. According to the diversity of extracellular lectin domains, lectin receptor protein kinases can be divided into L, G, and C types [10], in which the L and G types are plant-specific, while type C mainly exists in mammals [11]. It has been reported that *Arabidopsis thaliana*, rice, and soybean contain 77, 173, and 205 LecRLKs, respectively [12].

The phylogenetic tree of the members of the *GmLecRLKs* family in soybean has been constructed, and the classification of the members of the family in soybean is similar to that in *Arabidopsis thaliana*, which was also divided into G, L, and C types. There are 138 members of type G and 65 members of type L, while type C has only two members [13]. As important receptor proteins for signal reception, *LecRLKs* play important roles in the response to external stress and participate in hormone signaling pathways. A small number of members also play an important role in plant growth and development. For example, overexpression of the lectin receptor protein kinase gene *LecRK-V5* in *Arabidopsis thaliana* can inhibit the plant’s response to abscisic acid (ABA), thus affecting stomatal opening and closing [14]. In leguminous plants, the *PsLecRLK* gene is a salt stress resistance gene that enhances the viability of plant cells by isolating the flow of sodium ions and increasing ROS to improve the salt tolerance of plants [15]. In rice, salt stress can increase the activity of SIT1 protein kinase and phosphorylate its downstream effector proteins, MAPK3 and MAPK6. In addition, the SIT1 protein can also mediate ethylene production and the ethylene signal pathway induced by salt stress [16]. The *GsSRK* gene, a member of the G type of the *Arabidopsis* LecRLK family, can be highly expressed by ABA, high salt, and drought stress, and its overexpression line survives under high salt stress; the chlorophyll content and plant height were significantly higher than those of the WT [17]. However, there are few reports on the involvement of the *GmLecRlk* gene in the molecular mechanism of salt stress resistance in soybean.

In a previous study, we found that overexpression of *GmNFYB1* in *Arabidopsis thaliana* increased tolerance to abiotic stress [18], and the differentially expressed gene *GmLecRlk* was obtained by mining transcriptome data of WT and *GmNFYB1-ox* transgenic soybean, which encodes a G-type lectin receptor protein kinase. In this study, we demonstrated that the expression of the *GmLecRlk* gene was induced by NaCl. *GmLecRlk-ox* transgenic soybean was obtained by the cotyledon node transformation method, and it was found that *GmLecRlk-ox* transgenic soybean enhanced tolerance to salt stress and reduced the accumulation of active oxygen. It was also found that the overexpression of *GmLecRlk* in roots could improve the salt tolerance of soybean. It is further proved that *GmLecRlk* improves the salt tolerance of soybean by upregulating *GmERF3*, *GmbHLH30*, and *GmDREB2* and downregulating *GmGH3*.*6*, *GmPUB8*, and *GmLAMP1*. Our research reveals a new mechanism of salt resistance in soybean. Manipulation of these genes should facilitate improvements in salt tolerance in soybean.

## 2. Results

### 2.1. Expression Pattern of GmLecRlk

**Figure 1:**
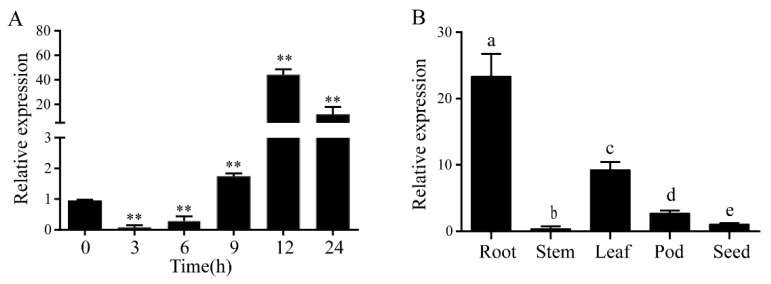
Expression pattern *of GmLecRlk*. (**A**) Expression analysis of *GmLecRlk* in response to salt stress by qRT-PCR. Asterisks indicate a significant difference compared with the corresponding control (Student’s *t* test: ** *p* < 0.01). (**B**) Tissue specificity analysis of the *GmLecRlk* gene. A one-way ANOVA was used to generate the *p* values. Error bars represent standard deviations. Values represent the means of three biological replicates.

Previous studies have shown that *LecRlk* genes are involved in many plants’ response to abiotic stress, such as *Arabidopsis thaliana* [14], *Oryza sativa* [16] and *Glycine soja* [17]. To explore whether the *GmLecRlk* gene responds to salt stress in soybean, expression patterns were identified using qRT-PCR. The results showed that the mRNA abundance of *GmLecRlk* decreased at first and then increased and reached the peak at 12 h, which was about 40 times that of the control (Figure 1A). Further analysis of its tissue specificity showed that it was expressed in the root, stem, leaf, pod, and seed, and the highest expression level was in the roots (Figure 1B).

### 2.2. GmLecRlk Improves Salt Tolerance in Soybean

**Figure 2:**
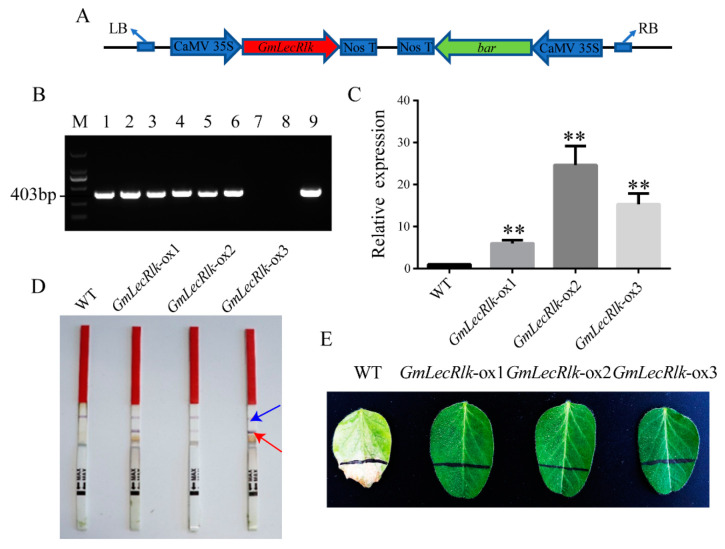
Identification of *GmLecRlk*-ox transgenic soybean. (**A**) Physical maps of the regions between left border (LB) and right border (RB); (**B**) gel image of PCR products obtained with primer sets for T-DNA regions of the vector. M: DL2000 marker. 1–6: positive plants 7: negative control (ddH2O). 8: DNA of WT soybean plants. 9: plasmid of the *pCAMBIA3300*-*GmLecRlk* vector. (**C**) *GmLecRlk* gene expression level in transgenic and WT soybean by qRT-PCR. Significant differences were analyzed based on the results of three biological replications (Student’s *t* test: ** *p* < 0.01). Bars indicate the standard error of the means (s.e.m). (**D**) Detection of the selectable marker gene bar by test strip. Blue arrow: control. Red arrow: bar is positive. (**E**) *GmLecRlk*-ox and WT soybean were screened by smearing 180 mg/L PPT on the initial leaves.

Expression pattern analysis showed that the *GmLecRlk* gene expression level was significantly induced by salt stress. We speculated that it may be involved in the tolerance of soybean to salt stress. To verify our conjecture, overexpressing *GmLecRlk* transgenic soybean (*GmLecRlk-ox*) was used for phenotypic observation under salt stress. T-DNA region schematic diagram of the carrier (Figure 2A). *GmLecRlk-ox* transgenic plants were identified by DNA and RNA levels (Figure 2B,C). The selectable marker gene bar was examined using a test strip and smeared PPT (glufosinate ammonium) (Figure 2D,E). The identified homozygous T3 seeds were used in subsequent experiments.

**Figure 3:**
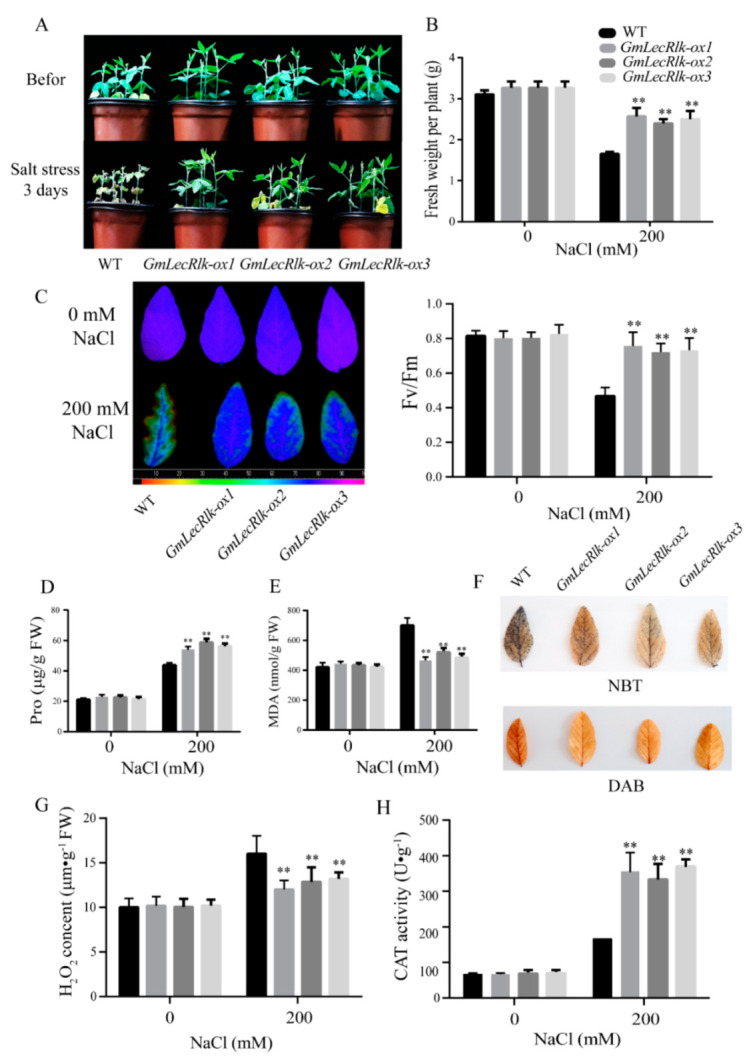
*GmLecRlk* improves salt tolerance in soybean. (**A**) Phenotypic analysis of *GmLecRlk*-ox and WT soybean under 200 mM NaCl; (**B**) fresh weight in *GmLecRlk*-ox and WT soybean with 0 or 200 mM NaCl; (**C**) Fv/Fm rate in *GmLecRlk*-ox and WT soybean with 0 or 200 mM NaCl. (**D,E**,**G**) Pro, MDA, and H_2_O_2_ content in *GmLecRlk*-ox and WT soybean with 0 or 200 mM NaCl. (**F**) *GmLecRlk*-ox and WT soybean leaves were stained with NBT and DAB under 200 mM NaCl for 3 days. (**H**) CAT activity in *GmLecRlk*-ox and WT soybean with 0 or 200 mM NaCl. **, *GmLecRlk*-ox soybean showed a significant difference from the WT (*p* < 0.01). Error bars, s.e.m.

After salt treatment for 3 days, the results showed that the leaves of the WT were seriously wilted and deformed, while most of the leaves of *GmLecRlk-ox* had been growing vigorously (Figure 3A). The fresh weight was measured after 3 days under salt stress, and the results showed that the fresh weight of *GmLecRlk-ox* was significantly higher than that of the WT (Figure 3B). The ratio of variable to maximal fluorescence (Fv/Fm) is an important parameter that reflects the photosynthetic status of plants under stress. Stress damages photosystem II and reduces the efficiency of Fv/Fm [19]. The Fv/Fm value of the *GmLecRlk-ox* transgenic soybean was significantly higher than that of the WT soybean after 2 days of salt stress (Figure 3C). The contents of Pro and MDA were further measured, and the results showed that the Pro content of *GmLecRlk-ox* plants was significantly higher than that of the WT, while the content of MDA was lower than that of WT plants (Figure 3D,E).

Previous studies have shown that *LecRlk* is involved in a variety of signal transduction pathways. It is necessary to further study whether *GmLecRlk* is involved in the ROS pathway. The results of NBT and DAB staining showed that *GmLecRlk-ox* plants accumulated less ROS than WT (Figure 3F). The measurement of H_2_O_2_ content and CAT activity further confirmed our idea. The results showed that the content of H_2_O_2_ of *GmLecRlk-ox* plants was significantly lower than that of the WT (Figure 3G), while the CAT activity was higher than that of WT plants (Figure 3H). The above results show that *GmLecRlk* improves salt tolerance in soybean and ROS scavenging ability.

**Figure 4:**
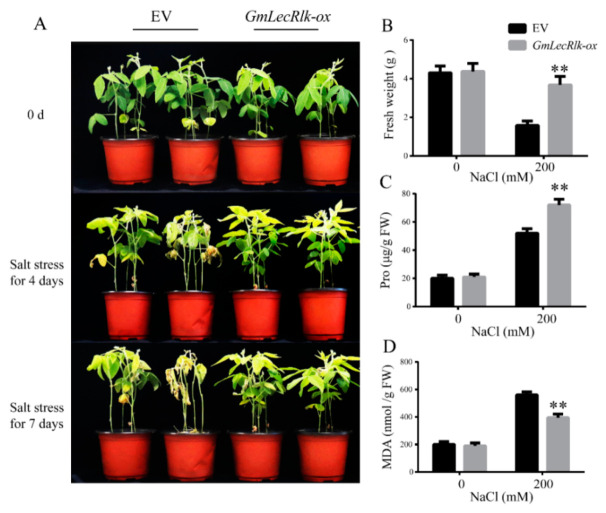
Phenotype identification of *GmLecRlk* under salt treatment in transgenic soybean hairy root compound plants. (**A**) Phenotypic analysis of *GmLecRlk*-ox and EV soybean hairy root compound plants grown under 0 or 200 mM NaCl; (**B**) fresh weight of *GmLecRlk*-ox and EV soybean hairy root compound plants with 0 or 200 mM NaCl after 7 days; (**C**,**D**) pro and MDA content in *GmLecRlk*-ox and EV soybean hairy root compound plants with 0 or 200 mM NaCl after 4 days. **, *GmLecRlk*-ox soybean hairy root compound plants showed a significant difference from EV (*p* < 0.01). Error bars, s.e.m.

Tissue-specific expression analysis showed that the expression of *GmLecRlk* was the highest in roots (Figure 1B). To verify whether its expression alone in roots could also improve the resistance to salt stress, soybean hairy root composite plants overexpressing *GmLecRlk* were used for further analysis. We first verified the feasibility of the method, and the *pCAMBIA3301-GUS* transgenic hairy root composite plants were obtained. It was found that hairy roots were dyed blue by GUS staining, which proved that the method was indeed effective (Figure S1A). The *GmLecRlk*-ox transgenic hairy root composite plants were identified by qRT-PCR (Figure S1B,C). After salt treatment for 4 days, the results showed that the soybean leaves of empty vector (EV) began to yellow and wilt, while the *GmLecRlk*-ox hairy root compound plants had no significant change. After 7 days, some plants in the control group began to die, while the leaves of *GmLecRlk*-ox hairy root compound plants turned yellow, but the soybean plants still survived (Figure 4A). The fresh weight of *GmLecRlk*-ox was significantly higher than that of EVs under salt stress (Figure 4B). Furthermore, we measured the contents of Pro and MDA in *GmLecRlk*-ox and EV soybean hairy root compound plants under salt stress for 4 days. The results showed that the Pro content of *GmLecRlk*-ox soybean hairy root compound plant leaves was significantly higher than that of EV, while the MDA content was less increased under 200 mM NaCl (Figure 4C,D). These results confirmed our hypothesis that the overexpression of *GmLecRlk* in roots alone could also improve soybean tolerance to salt stress.

### 2.3. Transcriptome Sequencing Analysis

**Figure 5:**
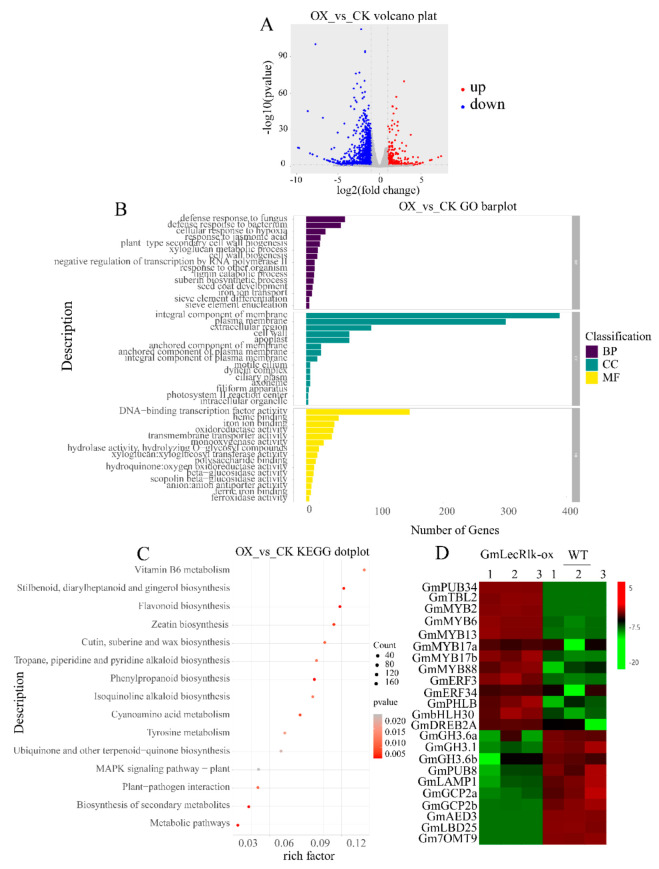
Transcriptomic analysis of *GmLecRlk-ox* and WT soybean. (**A**) Number of genes showing differential expression in *GmLecRlk-ox* and WT; (**B**) GO terms that were statistically enriched in differentially expressed genes in *GmLecRlk-ox* and WT; (**C**) KEGG enrichment analysis of differentially expressed genes in *GmLecRlk-ox* and WT plants; (**D**) The heat map of differential expression in *GmLecRlk-ox* and WT soybean. The numerical values for the green-to-red gradient bar represent the log2-fold change relative to the control sample.

To identify the potential downstream genes regulated by *GmLecRlk* in the salt stress response, we analyzed the transcriptome of *GmLecRlk-ox* transgenic soybean and the WT. The results showed that 1834 DEGs were identified in *GmLecRlk-ox* soybean compared with the WT under non-salt stress, of which 426 genes were upregulated and 1408 genes were downregulated (Figure 5A). The results of GO enrichment of differentially expressed genes showed that biological process (BP) was mainly involved in defense response to fungus, defense response to bacterium, cellular response to hypoxia, response to jasmonic acid, and plant type secondary cell wall biogenesis. The cellular component (CC) was mainly involved in integral components of the membrane, plasma membrane, extracellular region, cell wall, and apoplast. Molecular function (MF) was mainly involved in DNA-binding transcription factor activity, heme binding, iron ion binding, oxidoreductase activity, and transmembrane transporter activity (Figure 5B). The enrichment of the KEGG metabolic pathway showed that it was mainly involved in metabolic pathways, biosynthesis of secondary metabolites, plant–pathogen interaction, phenylpropanoid biosynthesis, and flavonoid biosynthesis (Figure 5C).

Many studies have demonstrated that some TF family members, including MYB, ERF, bHLH, and DREB, are involved in the response of plants to salt stress. In addition, some negative regulatory factors are also involved in plant salt stress response, such as indole-3-acetic acid-amido synthetase (GH3.6), glutamate carboxypeptidase (AMP1), and U-box domain-containing protein (PUB). Analysis of differential genes showed that 10 transcription factors were significantly upregulated: *GmMYB2* (G*lyma*.*09G183400*), *GmMYB6* (*Glyma*.*15G144000*), *GmMYB13* (*Glyma*.*09G038900*), *GmMYB17a* (*Glyma*.*09G261600*), *GmMYB17b* (*Glyma*.*10G236400*), *GmMYB88* (*Glyma*.*01G044300*), *GmERF3* (*Glyma*.*08G216600*), *GmERF34* (*Glyma*.*01G074200*), *GmbHLH30* (*Glyma*.*13G291400*), and *GmDREB2a* (*Glyma*.*12G103100*). Seven negative regulatory factors were significantly downregulated, including three indole-3-acetic acid-amido synthetase-family genes Gm*GH3*.*6a* (*Glyma*.*12G197800*), Gm*GH3*.*1* (*Glyma*.*05G101300*), Gm*GH3*.*6b* (*Glyma*.*13G304000*), and *GmPUB8* (*Glyma*.*15G064800*) and three glutamate carboxypeptidase-family genes Gm*LAMP1* (*Glyma*.*01G006700*), Gm*GCP2a* (*Glyma*.*03G205900*), and Gm*GCP2b* (*Glyma*.*10G088000*) (Figure 5D). Differential expression of these genes suggests that *GmLecRlk* may improve the salt tolerance of soybean by regulating these genes.

### 2.4. GmLecRlk Improves Salt Tolerance by Positively Regulating Salt Stress Response Genes

**Figure 6:**
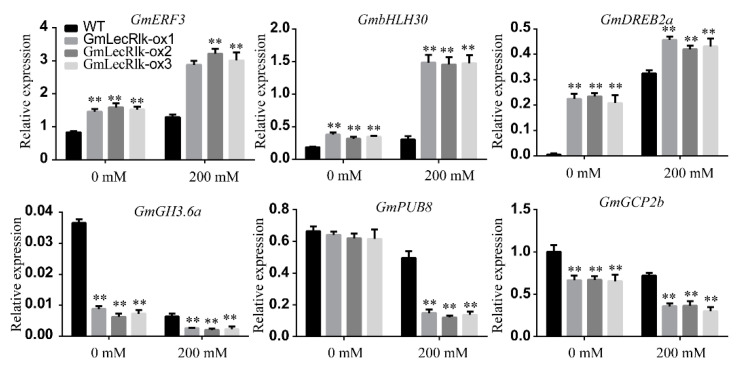
The transcriptional levels of *GmERF3*, *GmbHLH30*, *GmDREB2a*, *GmGH3*.*6a*, *GmPUB8*, and *GmGCP2* in *GmLecRlk-ox* and WT soybean under 0 or 200 mM NaCl for 12 h. **, *GmLecRlk*-ox soybean showed a significant difference from the WT (*p* < 0.01). Error bars, s.e.m.

Through the analysis of the data of differentially expressed genes in the transcriptome of *GmLecRlk-ox* and WT soybean, significant differences were observed in *GmERF3*, *GmbHLH30*, *GmDREB2a*, *GmGH3*.*6a*, *GmPUB8*, and *GmGCP2b*. To demonstrate the difference in the expression of these genes, we also measured the change in gene expression in *GmLecRlk-ox* and WT soybean by qRT-PCR after salt treatment for 12 h. The results showed that the *GmERF3*, *GmbHLH30*, and *GmDREB2a* genes were significantly upregulated, while *GmGH3*.*6a*, *GmPUB8*, and *GmGCP2b* were significantly downregulated in *GmLecRlk-ox* soybean (Figure 6). Moreover, we carried out qRT-PCR verification of *GmMYB6*, *GmMYB13*, and *GmMYB17b*, which had not been clearly reported for salt tolerance, and the most significant differences in transcriptome data were for *GmPUB34* (*Glyma*.*15G000700*), *GmTBL2* (*Glyma*.*18G140800*), *GmAED3* (*Glyma*.*02G213000*), *GmLBD25* (*Glyma*.*19G063600*), and *Gm7OMT9* (*Glyma*.*20G213600*). Compared with the WT, the expression of these genes was significantly different from that of *GmLecRlk-ox* soybean in 0 or 200 mM NaCl (Figure S2). These results suggest that *GmLecRlk* participates in the response to salt stress by regulating *GmERF3*, *GmbHLH30*, *GmDREB2a*, *GmGH3*.*6a*, *GmPUB8*, *GmGCP2b*, or some potential regulatory factors (*GmMYB6*, *GmMYB13*, *GmMYB17b*, *GmPUB34*, *GmTBL2*, *GmAED3*, *GmLBD25*, and *Gm7OMT9*).

## 3. Discussion

Soybean is an important oil crop, but salt stress significantly affects soybean yield. Therefore, the identification of some salt-resistant genes and the cultivation of salt-tolerant soybean varieties can improve the resistance of soybean to salt stress and increase the use of saline–alkali land. Recent studies have shown that the *S-locus LecRLK* gene of *Glycine soja* (*GsSRK*) enhances salt tolerance in *Arabidopsis* and alfalfa by increasing SOD activity and scavenging ROS [20]. *GmCrRLK1L20* can improve the salt tolerance of soybean by upregulating the expression of salt stress response genes, such as *GmMYB84*, *GmWRKY40*, *GmDREB-like*, *GmGST15*, *GmNAC29*, and *GmbZIP78* [21]. Similar to the above results, *GmLecRlk* can also improve soybean tolerance to salt stress, but its regulatory mechanism is not very clear.

Previous studies have reported that some ERF, bHLH, and DREB transcription factors are involved in the response of plants to salt stress [21,22,23]. For example, *GmERF3* overexpression in tobacco enhances salt resistance in plants by increasing free Pro and soluble carbohydrates [21]. The transgenic *GmbHLH30* gene improves plant tolerance to aluminum by increasing the content of soluble sugar and Pro in *N*. *benthamiana* [22]. *GmDREB2* enhances the salt tolerance of *Arabidopsis thaliana* and *N*. *benthamiana* by activating the expression of downstream salt-resistance genes [23]. Furthermore, some negative regulatory factors are also involved in the response of plants to salt stress, such as *AtAMP1,* which encodes a putative glutamate carboxypeptidase, and *amp1* mutants enhance salt tolerance by upregulating downstream salt-resistance genes *RD29A*, *AHA3*, and *ZAT10/12* in *Arabidopsis thaliana* [24]. Compared with the *pub23* mutant, *GmPUB8* overexpression enhanced salt tolerance by upregulating eight salt-related genes in *Arabidopsis thaliana* [25]. *AtGH3-6* encodes an indole-3-acetic acid-amidosynthetase that is induced by salt stress. Overexpressing *GH3-6* in *Arabidopsis thaliana* negatively regulates resistance to salt stress by inhibiting the expression of *RD22*, *KIN1*, *RD29A*, and *DREB1A* [26]. In this study, transcriptome analysis showed that two ERF-family genes, a bHLH-family gene, and a DREB-family transcription factor gene were significantly upregulated, and three AMP1-family genes, three GH3-family genes, and a PUB-family gene were significantly downregulated. Based on the combination of transcriptome and qRT-PCR analyses, we determined that *GmLecRlk* improved the salt tolerance of soybean by upregulating the expression level of salt response genes *GmERF3*, *GmbHLH30*, and *GmDREB2,* and downregulating the expression level of *GmGH3*.*6a*, *GmPUB8*, and *GmGCP2b*.

In addition, some MYB family transcription factors play an important role in plant responses to salt stress. *GmMYB84* enhances the salt tolerance of soybean by binding to the promoter of *GmAKT1* and upregulating the expression of *GmAKT1* [27]. *GmMYB68* was induced by salt stress and improved the salt–alkali tolerance of soybean [28]. *GsMYB15* [29], *GmMYB76*, *GmMYB92*, *GmMYB177* [30], *GmMYB81* [31], and *GmMYB12* [32] were regulated by salt stress and improved salt tolerance in *Arabidopsis thaliana*. Our results showed that the expression levels of *GmMYB6*, *GmMYB13*, and *GmMYB17b* were induced by salt stress and were significantly upregulated in *GmLecRlk*-*ox* soybean (Figure S2). Therefore, we speculated that *GmLecRlk* might also enhance the salt tolerance of soybean by regulating these genes.

In this study, we found for the first time that *LecRlk* was involved in soybean salt stress response. It was also proved that *GmLecRlk-ox* enhanced the tolerance of soybean to salt stress by scavenging reactive oxygen species and positively regulating salt stress response genes. Through pot cultural experiment, it was found that the plant height and yield of *GmLecRlk-ox* soybean were significantly higher than those of WT under salt stress (Tables S2 and S3). WT soybeans almost never harvest under salt stress, while *GmLecRlk-ox* soybeans maintain a high yield. Interestingly, tissue specificity analysis showed that the expression of *GmLecRLK* was the highest in soybean roots, but almost undetectable in stems and seeds. Further discovery of the overexpression of *GmLecRlk* in soybean hairy roots alone could also improve soybean tolerance to salt stress. This finding provides a new idea for the breeding of genetically modified soybeans. As a member of the RLK family, lectin receptor protein kinases can participate in biotic and abiotic stress response and plant development regulation. However, thus far, only a small number of lectin receptor protein kinases have been reported, and their interaction factors are little known. In the future, we will use yeast library screening and mass spectrometry to identify *GmLecRlk* interaction factors and phosphorylation regulatory network.

## 4. Materials and Methods

### 4.1. Plant Materials and NaCl Stress Treatment

Soybean stable transgenic lines were generated from the ‘DongNong 50′ (DN50) background. For salt stress treatment, *GmLecRlk-ox* and WT soybean seeds were planted in 10 cm × 10 cm round plastic pots (vermiculite: peat soil is 1:3, five seeds per pot) and cultured in a greenhouse under long days (16-h light/8-h dark), 25 °C, and 60% humidity. When the plant grew for 20 days, the same plants was selected to irrigate 200 mM NaCl. After 3 days, the phenotype was observed, and the fresh weight of each plant was measured.

### 4.2. Plasmid Construction and Plant Transformation

To obtain *GmLecRlk*-overexpressing soybean, the coding sequence (CDS) of *GmLecRlk* was amplified by PCR from DN50 total cDNA as a template with primers *GmLecRlk*-F/R (Table S1) and then ligated to a large fragment of the pCAMBIA3300 vector digested by *Xba*I and *BamH*I by homologous recombination (ClonExpress II One Step Cloning Kit, Vazyme, Nanjing, China) to generate *pCAMBIA3300-GmLecRlk*. The recombinant plasmids were transformed into *Agrobacterium rhizogenes*, EHA105. According to the method described previously [33], the *GmLecRlk*-overexpressing DN50 transgenic soybean was obtained. Genetically modified soybeans were screened by smearing 180 mg/L glyphosate on the initial leaves and further validated by PCR (the primer bar-F/R was used for PCR identification, Table S1) and qPCR (the primer q*GmLecRlk*-F/R was used for qRT-PCR analysis, Table S1). Finally, a bar test strip was used to identify bar screening markers. T3 homozygous lines were used for further analysis.

To obtain transgenic soybean hairy root compound plants, *pCAMBIA3301-GUS*, *pCAMBIA3300* and *pCAMBIA3300-GmLecRlk* were introduced into *Agrobacterium rhizogenes* strain K599. According to the method described previously [34], transgenic soybean hairy root compound plants were obtained and identified by qRT-PCR. When the transgenic plant complex grew to the third ternate leaf, 200 mM NaCl was used for irrigation. The phenotype was observed for 4 and 7 days, and the survival rate was counted on the 7th day under salt stress.

### 4.3. Chlorophyll Fluorescence Measurement

The maximum efficiency of photosystem II (PSII) photochemistry (Fv/Fm ratio) was measured by modulating a chlorophyll fluorometer (Max-PAM, Heinz Walz, Effeltrich, Germany). After 20-day-old *GmLecRlk-ox* and WT plants were exposed to salt stress for 2 days, the leaves were collected immediately and the Fv/Fm ratio was measured. All measurements were recorded from three biological replicates.

### 4.4. Measurements of Proline, Malondialdehyde, and H_2_O_2_ Contents

Twenty-day-old *GmLecRlk-ox* and WT soybean plants were irrigated with 200 mM NaCl. Two days later, the leaves were collected and immediately ground on ice. Pro, malondialdehyde and H_2_O_2_ contents were measured by a Pro amino acid content determination kit, a malondialdehyde content determination kit, and a H_2_O_2_ content determination kit (Nanjing Jiancheng, Nanjing, China), respectively. The method was based on the manufacturer’s protocols. All measurements were biologically repeated three times.

### 4.5. Quantitative PCR Analysis

For the analysis of the expression pattern under salt stress, the WT was placed in a black plastic bowl containing vermiculite 5 cm × 5 cm (two seeds per bowl), under long-day conditions in a greenhouse (16 h light/8 h dark), 25 °C, and 60% humidity. When the seedlings grew second ternate compound leaves, the vermiculite was washed from the roots with clean water, and they were balanced in distilled water for 24 h. Then, the seedlings with the same growth were equally divided into two groups, one in distilled water as a control and the other in water containing 200 mM NaCl as a treatment group. Leaf samples were collected at 0, 3, 6, 9, 12, and 24 h after salt treatment.

For plant tissue specificity analysis, roots, stems, and leaves were collected from the plant at the first trifoliate stage (V1), and pods and beans were collected at the mature stage (R6). All tissues were immediately frozen in liquid nitrogen and placed in a refrigerator at –80 °C for RNA extraction. The total RNA was extracted by RNAiso Plus (Takara, Beijing, China, 9108), and the cDNA chain was synthesized by the Prime Script RT reagent kit with gDNA Eraser (Takara, Beijing, China, RR047A), qRT-PCR was performed by using an ABI-7500 fast platform with the TB Green^®^ Fast qPCR Mix (Takara, Beijing, China, RR430A). The program was run under the following settings: pre-denaturation at 95 °C for 30 s, followed by a 40-cycle program (95 °C, 5 s; 60 °C, 15 s; per cycle). The soybean housekeeping gene *GmActin4* was used as the internal reference gene. The gene expression rate was calculated by the 2^−ΔΔCT^ method. All experiments were analyzed with three technical and three biological replications.

### 4.6. Transcriptomic Analysis

For transcriptomic analysis, trifoliate leaves from three-week-old WT and *GmLecRlk-ox* transgenic soybean plants under non-salt conditions were collected and frozen in liquid nitrogen and sent to biological companies. cDNA library preparation, RNA-seq sequencing, and assembly were performed on the Illumina Nova 6000 (Illumina, San Diego, CA, USA) sequencing platform. For identification of differentially expressed genes, log2 ratios were calculated with the reads per kilobase of exon model per million mapped reads (RPKM) value. Every gene with a *p*-value ≤ 0.05 and a fold change ≥2 was divided into upregulated and downregulated transcripts. GO annotation analyses were completed using BLAST2GO software (BioBam, Valencia, ES-Spain) (https://www.blast2go. com/ [accessed on 8 October 2021]), an automated tool for the assignment of GO terms.

### 4.7. Statistics of Agronomic Characters

*GmLecRlk-ox* transgenic and WT soybean were planted in 20 cm × 20 cm round plastic pots (peat soil, five seeds per pot). When the plant grew to the third compound leaf, it was treated with NaCl stress. Each pot was irrigated with 300 mL NaCl solution with a concentration of 200 mM, and the control treatment was irrigated with the same amount of Hoagland nutrient solution. According to the development status of soybean plants, Hoagland nutrient solution was irrigated regularly to maintain its growth after salt stress treatment. The agronomic characters were counted after maturity.

### 4.8. Statistical Analysis

At least three biological replicates were included in the data, and all data were analyzed using Student’s *t* test or analysis of variance (ANOVA) for the determination of the significant differences with SPSS 25.0 (IBM, Armonk, NY, USA). All data were analyzed using GraphPad Prism 8.0.2 (GraphPad Software, San Diego, CA, USA) for calculating the means and standard error of the mean.

## 5. Conclusions

In this study, we investigated the biological functions of the soybean *GmLecRlk* in response to salt stress. The results show that the *GmLecRlk* gene enhanced ROS scavenging ability in response to salt stress in soybean. Overexpression of the GmLecRlk gene also conferred salt tolerance in soybean when it was overexpressed alone in soybean hairy root. This provides new insight into the mechanism of plant roots responding to environmental stress signals. The combination of RNA-seq and qRT–PCR analysis demonstrated that *GmLecRlk* improves the salt tolerance of soybean by upregulating *GmERF3*, *GmbHLH30*, and *GmDREB2* and downregulating *GmGH3*.*6*, *GmPUB8*, and *GmLAMP1*. Our research reveals a new mechanism of salt stress resistance in soybean, which shows a new method for the cultivation of new salt-resistant varieties.

## Data Availability

The RNA-seq data names of the repositories and accession numbers are as follows: NCBI-SRA database under BioProject no. PRJNA788354 and accession Nos. SRR17222406, SRR17222407, SRR17222408, SRR17222409, SRR17222410, and SRR17222411.

## Supplementary Materials

The following are available online at https://www.mdpi.com/article/10.3390/ijms23031030/s1.
