# Supplementary material for: GmLecRlk, a Lectin Receptor-like Protein Kinase, Contributes to Salt Stress Tolerance by Regulating Salt-Responsive Genes in Soybean
Source: Int J Mol Sci. 2022 Jan 18;23(3):1030. doi: 10.3390/ijms23031030 (PMC8835537; doi:10.3390/ijms23031030)
Supplement: Supplementary file 1 [file ijms-23-01030-s001.zip › ijms-1548199-supplementary.pdf]

Table S1 primer list

Primer name	Primer sequence
GmLecRlk -F	TCTAGAATGGCTCTTGCTCCATCC
GmLecRlk -R	GGATCCGCATCTTCCTAATCAAGAAGTC
qGmLecRlk -F	ATTTTTGAACCGTTTGAGGAGG
qGmLecRlk -R	CCCTGTCAAAGTATGCAAACAA
GmActin4-F	GTGTCAGCCATACTGTCCCCATTT
GmActin4-R	GTTTCAAGCTCTTGCTCGTAATCA
Bar-F	GCGGTACCGGCAGGCTGAAG
Bar-R	CCGCAGGAACCGCAGGAGTG
GmMYB2 -F	TGGAGTGTTGAAGAGGATGATC
GmMYB2 -R	GCTCTTCCGAGGTTAAATTTCC
GmMYB6 -F	AAAACCTTGCCTAAGAATGCAG
GmMYB6-R	CCTATTACCAAGAACAGCATGC
GmMYB13-F	CAAGAATGCAGGTTTGTTGAGA
GmMYB13 -R	CCTATTACCAAGAACAGCATGC
GmMYB17a -F	GCTGCAATAGCCTCTCAATTAC
GmMYB17a -R	TTTTGAGACGCTTCTTCAAGTG
GmMYB88 -F	GAAGCATTAGCAAACGAGAACA
GmMYB88 -R	TATGCGATCTGTCTCCAAAGTT
GmERF3 -F	GCTAGGAACTTTTGAAACAGCA
GmERF3 -R	GTGACACTTATGTAACTTGGCC
GmERF34 -F	CATCGTCATCACCAACATCTTC
GmERF34 -R	CTTGCTATTTGGGGTGTTTTGA
GmbHLH30 -F	CAAACCACCTTTGTCACTTCAT
GmbHLH30 -R	GAAAGTGAAGGAGAAGCTTTGG
GmMYB17b-F	AGGAGTAGTGAGTCAAAGCCTTG
GmMYB17b-R	CTCCAGCTTTGGAATGCAACTAC
GmDREB2A-F	CTTTTTCCGAGGAACCGTCTAGA
GmDREB2A-R	CTCCACCAGAATTGTTCCACAAC
GmGH36(1) -F	" TACAAAAGCTCCCATTTCAAGC"
GmGH36(1) -R	CTCGAAGAACTTCCTCGTTTTG
GmGH31 -F	TGTGCACACGTTTAATTAGTCG
GmGH31 -R	TGAAAGTTACATGCACGTGAAG
GmGH36(2) -F	CAAAAGCTCACATTTCAAGCAC
GmGH36(2) -R	GGTAAAGGCCACAAAGTAACTG
GmPUB8-F	CAGTGAAGAAACTGTTGTCGAG
GmPUB8-R	TTCCTCTTGACCTTGGCATTAT
GmLAMP1-F	AGTAGTAGTGGTGATGGAGAGT
GmLAMP1-R	TCATTATCTTCTCCCCATCAGC
GmGCP2a-F	TTTACGGCGAGGGTGACA
GmGCP2a-R	GAGTAAGTGGGTCTCCTATCCC
GmGCP2b -F	AAGCTGTAGCATACCTCAATGT
GmGCP2b-R	TTGACCTGTTTTATGACCTCGA

GmAED3-F	GACCGGTTTACAGTGCAATAAG
GmAED3-R	GCGTCAAATTCTCGTAGGTTTT
GmLBD25-F	CTTGGGGATGGTTATCACAGAG
GmLBD25-R	CACACTATGACTATACCACCCC
Gm7OMT9-F	GCCAAAATTATCTGTGAGGCAT
Gm7OMT9-R	CATATCACCACCGACAAAACTC
GmPUB34-F	ATATTCATCACAGAGCGTGGAT
GmPUB34-R	GTACATTTCCATCCTTTGCGTT
GmTBL2-F	AATTCTTCCCATTGGTTTGAGC
GmTBL2-R	TCAAAGTACTATGACACGTGCT

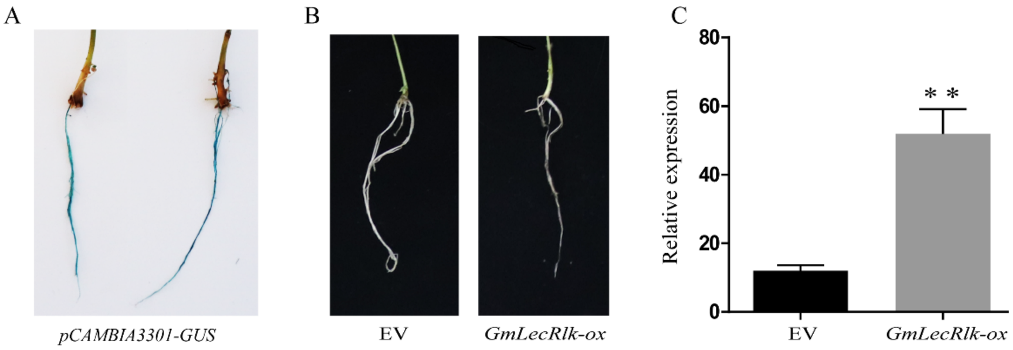

Figure S1. Identification of transgenic hairy roots. (A) GUS staining of *pCAMBIA3301-GUS* transgenic hairy roots. (B) Image of EV and *GmLecRlk-ox* transgenic hairy roots. (C) The expression level of *GmLecRlk* in transgenic soybean hairy root compound plants. *GmActin4* is used as the internal reference gene. The data shown are the means of three independent repeated experiments ± standard deviation. A significant difference between *GmLecRlk* and EV soybean hairy root compound plants is indicated by asterisks (** $P < 0.01$).

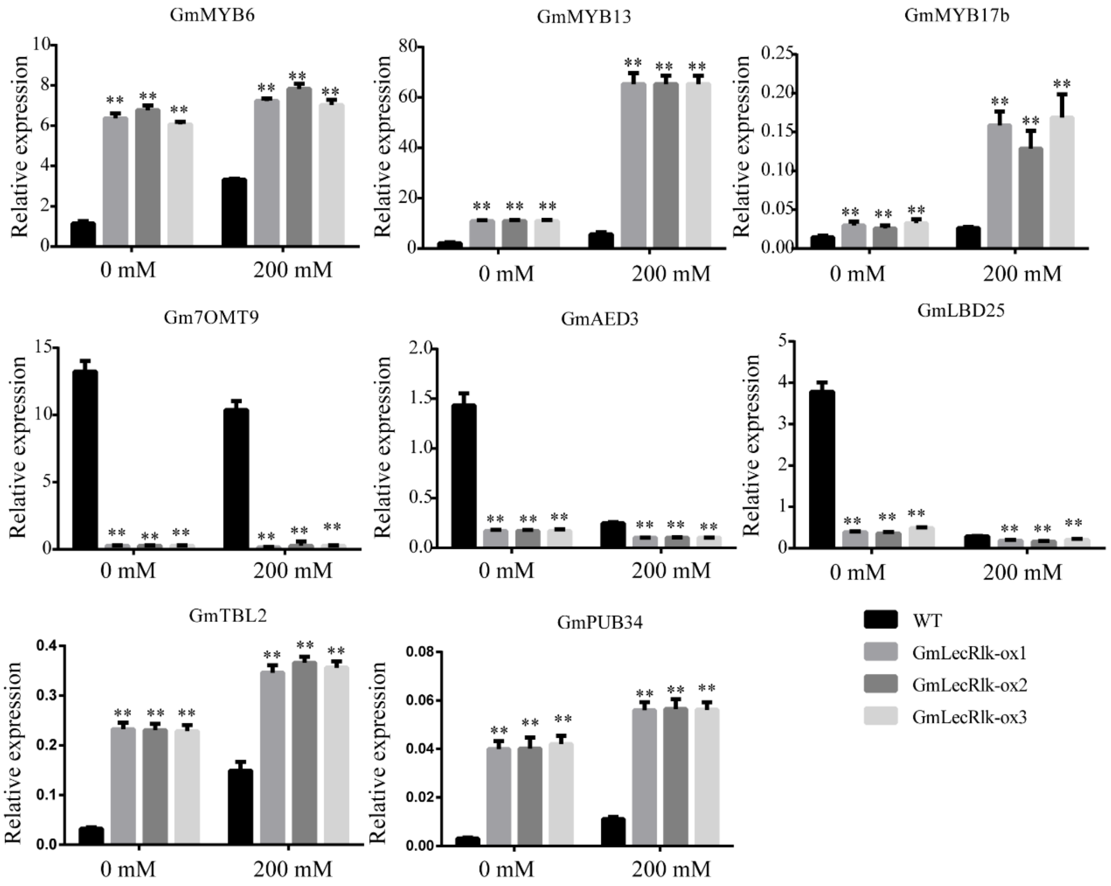

Figure S2. The transcription levels of *GmMYB6*, *GmMYB13*, *GmMYB17b*, *Gm7OMT9*, *GmAED3*, and *GmLBD25*. *GmTBL2* and *GmPUB34* in *GmLecRlk-ox* and WT soybean under 0 mM or 200 mM NaCl for 12 h. **, *GmLecRlk*-ox soybean showed a significant difference from the WT ($P < 0.01$). Error bars, s.e.m.

Table S2 Agronomic characteristics under non-salt stress

genotype	Plant/pot	Average plant height	Average seed number/pot	Average seed weight
WT	5	65.01±5.51	230.63±88.63	19.12±7.79
GmLecRlk-ox1	5	70.33±9.82**	292.38±82.27**	26.34±6.08**
GmLecRlk-ox2	5	74.45±5.25**	312.86±48.98**	26.33±3.50**
GmLecRlk-ox3	5	73.33±0.47**	296.75±46.51**	29.87±3.51**

**, *GmLecRlk*-ox soybean showed a significant difference from the WT ($P < 0.01$). Error, s.e.m

Table S3 Agronomic characteristics under salt stress

genotype	Plant/pot	Average plant height	Average seed number/pot	Average seed weight
WT	5	24.40±12.40	5.86±1.40	0.45±0.13
GmLecRlk-ox1	5	59.80±9.77**	131.89±31.35**	7.47±1.71**
GmLecRlk-ox2	5	51.48±4.12**	135.13±43.61**	7.61±2.12**
GmLecRlk-ox3	5	54.40±12.40**	138.8±40.75**	6.75±2.01**

**, *GmLecRlk*-ox soybean showed a significant difference from the WT ($P < 0.01$). Error, s.e.m
